# A method for modelling polymer electrolyte decomposition during the Li-nucleation process in Li-metal batteries

**DOI:** 10.1038/s41598-023-36271-5

**Published:** 2023-06-04

**Authors:** Liang-Ting Wu, Edvin K. W. Andersson, Maria Hahlin, Jonas Mindemark, Daniel Brandell, Jyh-Chiang Jiang

**Affiliations:** 1 Department of Chemical Engineering, National Taiwan University of Science and Technology, Taipei, 106 Taiwan; 2 Department of Chemistry - Ångström Laboratory, Uppsala University, Box 538, 75121 Uppsala, Sweden; 3 Department of Physics and Astronomy, Uppsala University, Box 516, 75120 Uppsala, Sweden

**Keywords:** Batteries, Theory and computation

## Abstract

Elucidating the complex degradation pathways and formed decomposition products of the electrolytes in Li-metal batteries remains challenging. So far, computational studies have been dominated by studying the reactions at inert Li-metal surfaces. In contrast, this study combines DFT and AIMD calculations to explore the Li-nucleation process for studying interfacial reactions during Li-plating by introducing Li-atoms close to the metal surface. These Li-atoms were added into the PEO polymer electrolytes in three stages to simulate the spontaneous reactions. It is found that the highly reactive Li-atoms added during the simulated nucleation contribute to PEO decomposition, and the resulting SEI components in this calculation include lithium alkoxide, ethylene, and lithium ethylene complexes. Meanwhile, the analysis of atomic charge provides a reliable guideline for XPS spectrum fitting in these complicated multicomponent systems. This work gives new insights into the Li-nucleation process, and experimental XPS data supporting this computational strategy. The AIMD/DFT approach combined with surface XPS spectra can thus help efficiently screen potential polymer materials for solid-state battery polymer electrolytes.

## Introduction

Lithium-metal batteries are promising candidates for next-generation energy storage devices owing to the low reduction potential and high theoretical capacity (3862 mAh g^−1^) of Li-metal^1–3^. However, the highly reactive Li-metal brings risks of dendrite formation and electrolyte depletion when the Li-metal reacts with volatile organic solvents in common liquid electrolytes^4,5^. The solid-state polymer electrolyte (SPE) constitutes one of the solutions for practical applications^6–9^. The non-flammable polymer promotes battery safety during operation^10^, and the excellent flexibility increases the interfacial compatibility between the Li-metal anode and the solid-state electrolyte as compared to ceramic counterparts. The low fabrication cost also benefits the mass-production of solid-state polymer electrolytes. Designing a novel polymer electrolyte that would produce a stable solid electrolyte interphase (SEI) layer with high ionic conductivity to raise the battery performance is one of the supreme goals of this field^11–14^. However, little is still known about the formation mechanism and the composition of the SEI layer. Therefore, an atomistic understanding of the complicated interfacial reactions between anode and electrolyte is essential^15–21^.

Poly(ethylene oxide) (PEO) has been widely investigated as the polymer host used in SPE applications^22,23^. Due to strong O-Li binding energy, the ether oxygen provides good solvation sites for the Li-ions dissolved from Li salts. Recently, computational results^24^ demonstrated that lithium oxide (Li_2_O) formation via cleavage of the PEO C-O bonds is highly thermodynamically favorable. In contrast, in X-ray photoelectron spectroscopy (XPS) studies^25,26^, lithium alkoxides (ROLi) were predominantly observed instead of lithium oxide during Li deposition. Moreover, in recent ab initio molecular dynamics (AIMD) simulation results^27^, PEO displayed high interfacial stability during the simulation when PEO was placed on a Li (100) surface. Thereby, such AIMD result are not fully consistent with experimental results, which motivates the search for novel computational approaches.

In this work, we continue to investigate the decomposition mechanism of PEO on the Li (100) anode surface. The difference in reactivity between adding Li-atoms to the system versus employing a fixed surface is explored. The addition of Li-atoms is meant to mimic the lithium nucleation process in a lithium metal cell, in a way similar to the in situ lithium deposition experiments performed by our group^26^. In that experiment, additional Li atoms were deposited into the SPE film, which played a role in the degradation of the SPE. The computational approach for Li-nucleation was inspired by this experimental setup. Charge analysis is then used, as employed in our recent study on PCL^28^, to connect the PEO spectra from the deposition study to the computational results achieved here, as a more positive charge means higher binding energy for the core electrons, and vice versa.

## Results

**Figure 1:**
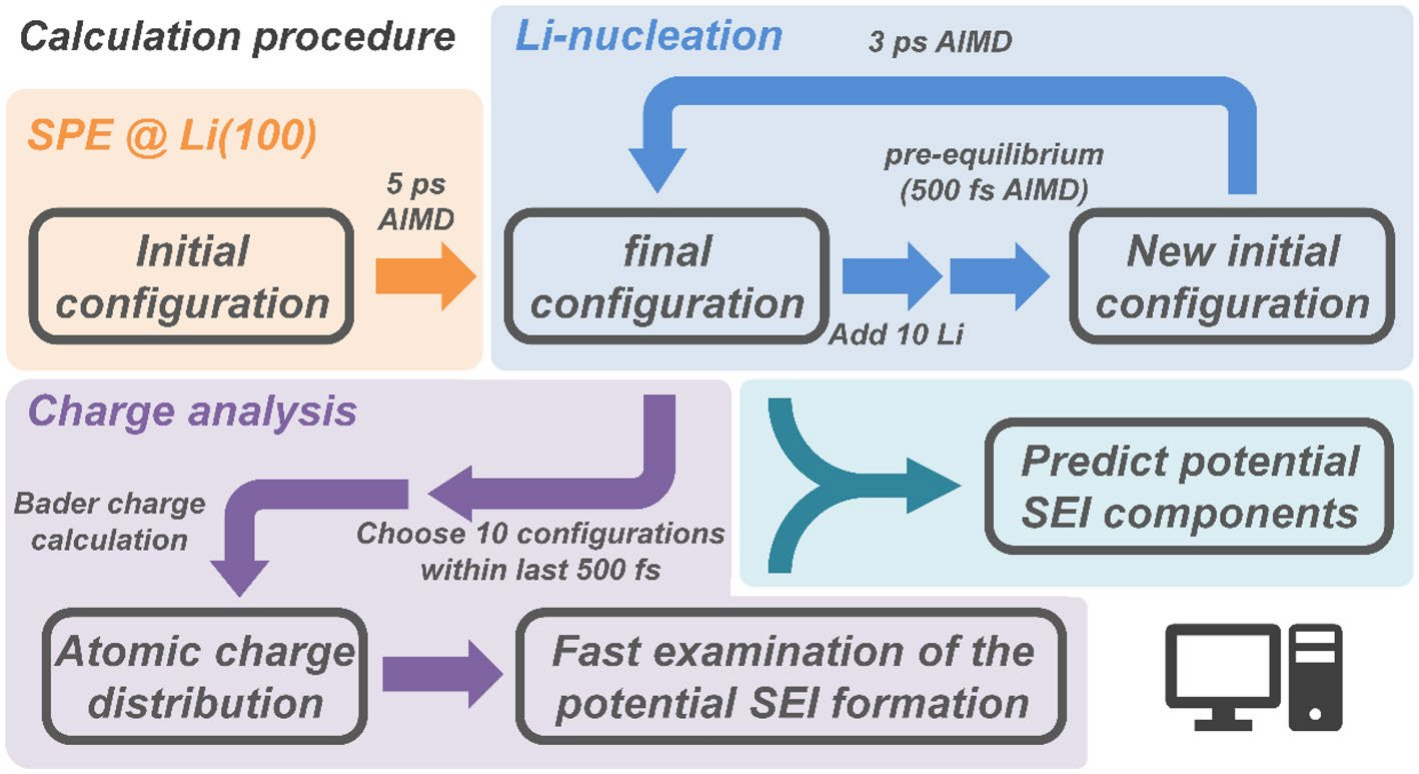
Overall calculation procedure of predicting potential SEI components.

The flowchart for the simulation of the PEO-lithium anode system is shown in Fig. 1. The overall calculations were carried out in four steps. First, a 5 ps AIMD simulation of SPE on the surface of the Li anode was performed. Then, 10 Li-atoms were added and pre-equilibrated for 500 fs to find reasonable initial positions for these while the rest of the system was fixed, and a subsequent 3 ps AIMD simulation was performed. This mimics the Li^+^ reduction during Li-plating. Li-atom addition and simulation were repeated two more times, for a total of 14 ps AIMD calculations, adding a total of 30 Li-atoms. The temperature was set to 600 K during pre-equilibrium to accelerate the relaxation of the added Li-atoms. After the pre-equilibrium of each stage, the polymer and the top three layers of the Li anode surface were relaxed, and the AIMD simulation of the PEO-Li anode system could proceed. The electronic charges were calculated using Bader charge analysis^29–31^. Ten configurations were selected within the last 500 fs close to the energy minimum to obtain the atomic charge at each step. Atoms from the different chemical environments were distinguished, and atomic charges were counted. The distribution of atomic charge for each chemical environment was presented by a Gaussian function.

**Figure 2:**
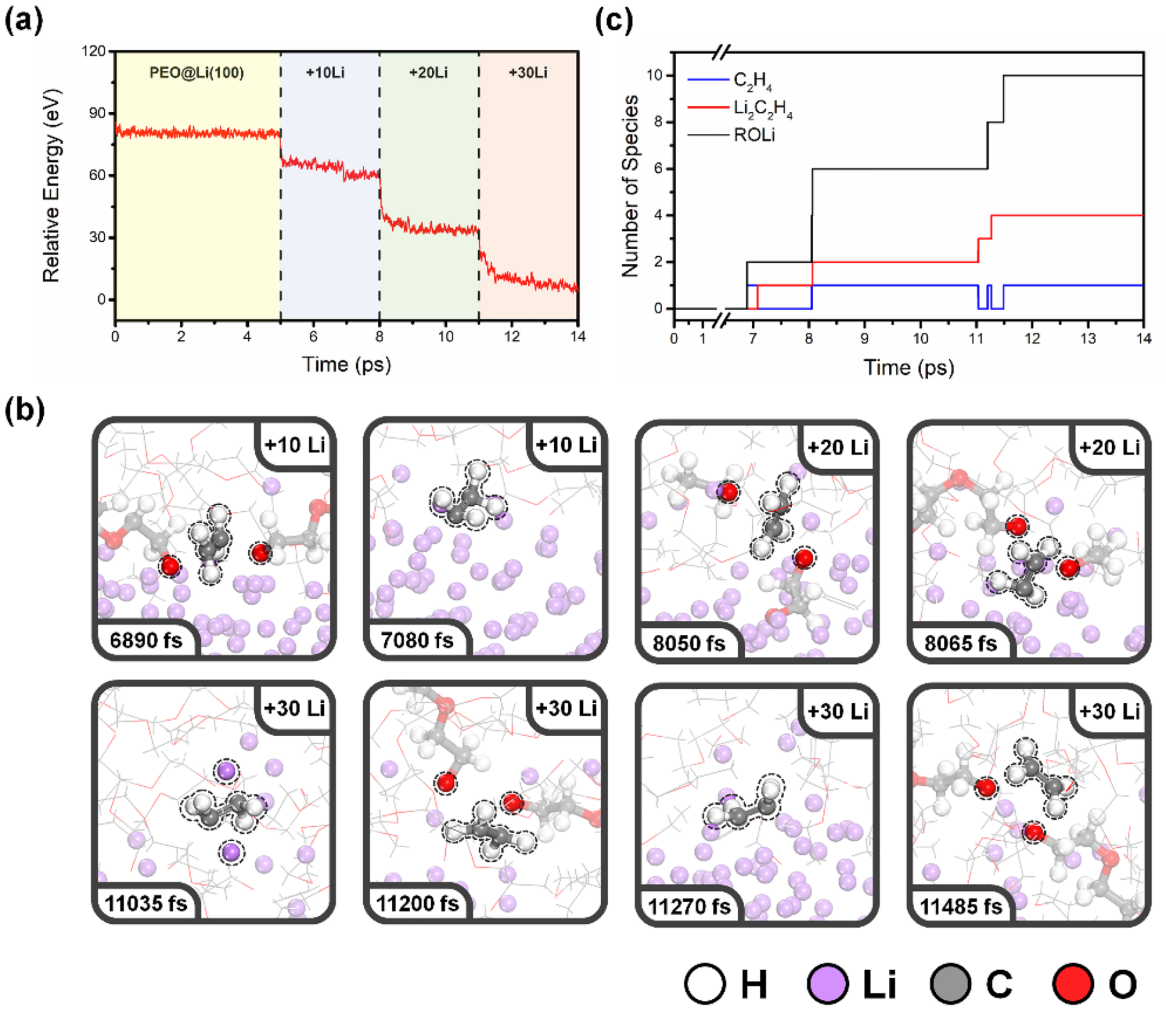
(**a**) Energy plot over the simulation time for PEO on Li (100) anode surface during Li-nucleation. (**b**) Snapshots of the PEO on Li (100) anode surface from AIMD simulations. (**c**) Number of degradation products obtained during decomposition reactions of PEO on the Li anode surface.

Figure S1 displays the AIMD simulation energy profile of the pure PEO. The components of the PEO did not undergo any degradation during the 10 ps AIMD simulation, and the system reached apparent thermal equilibrium within 1 ps. To understand the PEO decomposition mechanism observed in previous in situ XPS measurements^26^, in which the binding energy is calibrated using the 286.7 eV peak in the C 1*s* spectrum of PEO as the reference, we performed AIMD simulations in four stages, as described in Fig. 1. The energy plot of the PEO on the Li-anode system over the simulation time is shown in Fig. 2a. The first stage (yellow regions) represents the PEO on a Li (100) anode surface; the energy reaches apparent thermal equilibrium immediately, which implies that PEO decomposition did not occur at these time scales. After 5 ps simulation, we simulated the Li-nucleation process in three additional stages (blue, green, and red regions). Ten extra Li-atoms were added to the final configuration of the previous stage. An additional pre-equilibrium procedure was performed, and the energy fluctuation is shown in Fig. S2. For each Li-nucleation stage, the system reaches apparent thermal equilibrium within a 3 ps AIMD simulation, and the energy drops within each stage, implying that PEO decomposition has occurred. The simulation indicates that Li-reduction and -deposition trigger significantly more PEO decomposition than just exposing a PEO-based electrolyte to a Li-metal surface.

A series of snapshots at different simulation times extracted from the AIMD simulation are shown in Fig. 2b. The first PEO decomposition reaction was observed at 6890 fs, at the first Li-nucleation stage. The 1st PEO chain was decomposed, forming two lithium alkoxides, one of the major SEI components observed for PEO decomposition on the Li surface^25,26^. In addition, one ethylene molecule could be formed via two C–O bond cleavages at 7080 fs. The formed high-reactivity ethylene molecule interacted with the Li-metal surface and created lithium ethylene complexes (Li_2_C_2_H_4_)^32^. A sequence of PEO decomposition reactions was then observed at 8050, 8065, and 11,200 fs, and the produced ethylene molecules subsequently formed Li_2_C_2_H_4_ at 11,035, 8065, and 11,270 fs, respectively. The last PEO decomposition occurred at 11,485 fs, where the ethylene molecule remained in the system displaying *sp*^2^ hybridization until the end of the AIMD simulation. All the interfacial reactions and approximate times for events are summarized in Table S1. The resulting degradation products include lithium alkoxides (ROLi), ethylene molecules (C_2_H_4_), and Li_2_C_2_H_4_, forming the degradation species at the PEO-Li anode interface. The number of these species is illustrated in Fig. 2c, in which lithium alkoxide and Li_2_C_2_H_4_ are major SEI components. Ethylene molecules are usually formed intermediately, but are in the presence of Li^0^ immediately lithiated to Li_2_C_2_H_4_. It is important to note that AIMD simulations only qualitatively explore potential decomposition products and possible reaction mechanisms and provide the relative rather than absolute timing of the reactions.

**Figure 3:**
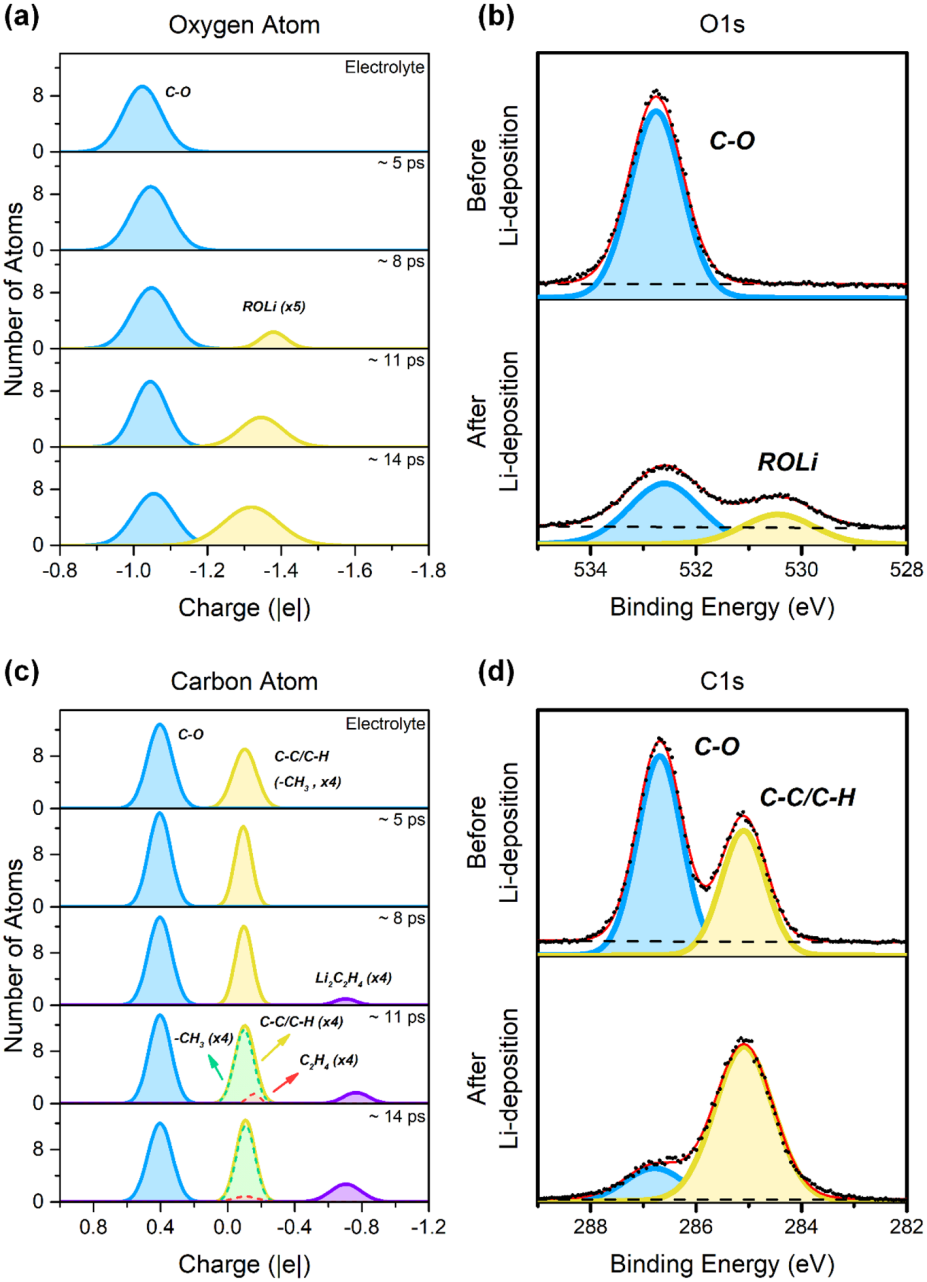
Atomic charge distribution of (**a**) oxygen and (**c**) carbon atoms in pure PEO and on Li (100) anode surface at different Li-nucleation stages. Reference (**b**) O 1*s* and (**d**) C 1*s* XPS spectra for pure PEO before and after Li-nucleation, using data previously published in Ref.^26^.

The atomic charge distribution of oxygen and carbon atoms in the PEO and PEO-Li anode system at different Li-nucleation stages is presented in Fig. 3a and c, respectively. For the oxygen atoms, only one chemical environment exists in the pure PEO, i.e., the ether oxygen (O–C, ~ − 1.02 |e|). After placing the PEO on the Li-anode surface, the distribution almost remains the same, but the center of the peak is shifted slightly towards more negative values (~ − 1.05 |e|), which is due to the oxygen atoms of the PEO chain being adsorbed on the Li-anode surface, causing the atomic charges of the oxygen atoms to become more negative. This downshift was also observed in the O 1*s* XPS spectrum of the PEO, where a shift in peak position from 532.7 to 532.6 eV was seen as the lithium was deposited on the PEO surface. An additional peak was observed in the range − 1.3 to − 1.4 |e| that indicates a new species formed, corresponding to lithium alkoxide (ROLi), while the oxygen atoms gain in electron density from the lithium metal. At the same time, a new peak in the experimental O 1*s* XPS spectrum at 530.4 eV verifies the formation of lithium alkoxide species. The intensity of the lithium alkoxide peak gradually increases after the following two Li-nucleation processes in the simulation. Two peaks were presented in the pure PEO system for the carbon atoms. The main peak ~  + 0.4 |e| corresponds to carbon atoms in the PEO backbone (C–O), which also were observed in C 1*s* XPS spectra at 286.7 eV. The small peak at ~ − 0.1 |e| corresponds to the end of the PEO chain (–CH_3_), which has the same chemical environment as the hydrocarbons (C–C/C–H), and the experimental C 1*s* XPS spectrum signal at 285.1 eV which corresponds to the surface hydrocarbon contamination in the sample preparation. In the simulations, the concentration of –CH_3_ groups is considerably higher than in the experimental counterparts, due to practical reasons. During the Li-nucleation processes, two new peaks were founded at ~ − 0.1 |e| and ~ − 0.7 |e|; the corresponding chemical environments are C_2_H_4_ and Li_2_C_2_H_4_ species, respectively. Considering the similar peak position of (–CH_3_) and C_2_H_4_, these would likely merge and appear as a single peak but with a small broadening, as seen from the growth of the C–C/C–H peak in Fig. 3d. Previous literature^21,25^ shows the presence of water could influence electrolyte decomposition. However, the absence of a LiOH peak in Fig. 3b demonstrates the water content is negligible, which can be attributed to the high vacuum condition in the XPS measurement (~ 10^–10^ mbar) and Li-deposition experiment (~ 10^–8^ mbar). As stated above, the atomic charge distribution is an efficient descriptor for understanding peaks in the XPS spectrum.

To investigate the role of extra Li in detail, we placed a PEO oligomer in a vacuum layer and adsorbed it on the Li (100) surface. We also adsorbed a PEO-Li complex on the Li (100) surface, which includes 5 layers of Li with a 5 × 5 supercell. The optimized structures of PEO before and after adsorption on the Li (100) surface, and a PEO-Li complex adsorbed on the Li (100) surface are shown in Fig. S3. The corresponding projected density of states (PDOS) of PEO in each system is shown in Fig. S4. After PEO adsorption on the Li surface, its conduction band, located above the Fermi level, highly hybridized with the Li surface and broadened to cover the Fermi level, which explains the electron transfer from the Li surface to the PEO. Moreover, the conduction band of the PEO-Li complex further downshifted when it adsorbed on the Li (100) surface. The downshift of the PEO conduction band after complexation with Li implies that the extra Li facilitates electron transfer from the Li surface to PEO. This is verified by the calculated charge of PEO. Initially, PEO carries zero charges in a vacuum layer. After adsorption on the Li surface, it gains electrons from the Li surface, resulting in a Bader charge of − 0.56 |e| for PEO. Furthermore, upon complexation with Li, it exhibits an even stronger tendency to receive electrons from the Li surface, leading to a Bader charge of − 0.74 |e| for PEO. Since the PEO-Li complex has a lower conduction band, the complex exhibits higher reactivity than pure PEO oligomer and undergoes reductive decomposition during the AIMD simulation.

However, the nature of these extra Li-atoms remains unclear. Therefore, we replaced the extra Li-atom with an extra Li-ion, forming a PEO-Li^+^ complex ion that adsorbed onto the Li (100) surface, as depicted in Fig. S5a. We found that the structures of PEO-Li and PEO-Li^+^ were almost identical when they were adsorbed on the Li (100) surface. The geometry of the PEO-Li @ Li (100) and PEO-Li^+^ @ Li (100) systems is represented by black and red ball-and-stick models, respectively, in Fig. S5b. Furthermore, the calculated Bader charge of PEO is − 0.74 |e| and − 0.67 |e| in the PEO-Li @ Li (100) and PEO-Li^+^ @ Li (100) systems, respectively. The extra Li in both systems exhibits a Bader charge of + 0.88 |e|, indicating its Li-ion character. This indicated that extra Li immediately transfers electrons to the PEO chain, forming Li-ions and excess electrons. The complexation of Li-ions with PEO lowers the energy of the PEO conduction band, while excess electrons fill in the low-lying PEO conduction band. Even though the PEO-Li^+^ @ Li (100) system carries a positive charge, the PEO still receives electrons from the Li surface since the Li-metal anode acts as the electron source. Therefore, the influence of the excess electron is negligible. The role of coordinated Li atoms in liquid electrolytes has been extensively studied in computational studies^33–35^, and these findings are also applicable to solid polymer electrolytes. Given this, the simulation approach used in this study can also be applied to model interfacial decomposition in real all-solid-state Li-metal batteries, as Li-ions and electrons transfer to the anode surface via the electrolyte and external circuits, respectively. The processes of PEO-Li complexation and excess electron transfer to PEO are essential in both Li-deposition and Li-metal battery charging.

## Discussion

In this study, we have thus established a computational strategy for simulating PEO decomposition on the Li-metal anode surface during the Li-nucleation process, which has combined AIMD simulations and DFT calculations to identify the resulting SEI components. The single Li-atom resulting from the reduction of Li-ions exhibits higher reactivity than the ideal and defect-free metal Li-anode commonly employed in similar calculations, owing to the different chemical environments. This indicates that taking the Li-nucleation process into account is crucial for estimating electrolyte degradation during Li-plating. We also proposed reasonable PEO decomposition pathways by AIMD simulation using this approach. It is then confirmed that the degradation species mainly comprises lithium alkoxide, ethylene, and Li_2_C_2_H_4_, consistent with previous XPS experimental results from the deposition study. Moreover, the calculated atomic charge distributions are highly in agreement with the XPS spectra, indicating that these calculations can aid the curve fitting of XPS spectra. Furthermore, Li salts are a necessary component for the polymer electrolyte. Degradation products of anions also contribute to unique interfacial phenomena in various polymer electrolytes. In the future, including Li salts in simulations are vital to helping polymer electrolyte screening.

## Methods

All DFT calculations in this study were performed with the projector augmented wave (PAW) method^36^ using the Vienna ab initio Simulation Package (VASP)^37,38^. The generalized gradient approximation (GGA) functional^39^ of Perdew-Burke-Ernzerhof (PBE) was used to describe exchange and correlation interactions. The DFT-D3 method was considered for including the van der Waals correction^27^. A plane wave cutoff energy of 500 eV was used for all calculations. The Brillouin zone was sampled with the gamma point. The electronic energy convergence was set to 10^−4^ eV for self-consistent iteration, and the force convergence was set to 0.01 eV/Å for ionic relaxation. The 5 × 5 Li (100) anode surface consists of seven layers, and the bottom two layers were replaced by He atoms to avoid interactions between periodic cells. In the z-direction, a vacuum layer of ~ 15 Å was added above the Li metal surface, and the electrolyte could be placed on the surface. The bottom four layers, including two layers of He and two layers of Li, were fixed during the simulations. The pure electrolyte system was equilibrated at 400 K in the canonical ensemble (NVT) for 10 ps with a time interval of 1 fs. In this study, the polymer in the electrolyte was poly(ethylene oxide) (PEO). The employed PEO oligomer (containing six repeat units), the simulation box of pure PEO electrolyte (containing ten PEO chains), and the simulation box of SPE on a Li (100) anode surface are shown in Fig. S6. The simulation box size of the pure PEO electrolyte system was 16.25 × 16.25 × 16.25 Å, and the size of the electrolyte-Li anode system was set to 16.91 × 16.91 × 26.03 Å, corresponding to a density of PEO electrolyte of 1.14 g cm^−3^. Before we performed the AIMD simulation of the PEO|Li (100) interfacial reaction, we conducted a classical MD pre-equilibrium using the COMPASS II force-field for 10 ps before the AIMD simulation (NVT ensemble at 400 K), just as we did in our previous study^28^. In the Li-nucleation process, we added extra Li atoms randomly to the PEO|Li system near the interface region. However, the location of the extra Li atoms can significantly affect the simulation results. Therefore, we performed a pre-equilibrium process to minimize this effect. We fixed all the atoms except the extra Li atoms, which ensured that no reactions or decomposition occurred during the pre-equilibrium process. Additionally, we used a high temperature of 600 K to provide the extra Li atoms with sufficient kinetic energy to accelerate the Li atoms into an energy-minimum location instead of the initial position we added.

## Supplementary Information

Supplementary Information 1.

Supplementary Information 2.

## Data Availability

All data generated or analyzed during this study are included in this article, and the datasets used or analyzed during the current study are available from the corresponding author with reasonable request.
