# Supplementary material for: A method for modelling polymer electrolyte decomposition during the Li-nucleation process in Li-metal batteries
Source: Sci Rep. 2023 Jun 4;13:9060. doi: 10.1038/s41598-023-36271-5 (PMC10239748; doi:10.1038/s41598-023-36271-5)
Supplement: Supplementary file 1 — Supplementary Information 1. [file 41598_2023_36271_MOESM1_ESM.docx]

**Supplementary Information**

**A Method for Modelling Polymer Electrolyte Decomposition During the Li-Nucleation Process in Li-Metal Batteries**

Liang-Ting Wu ^a,b^, Edvin K. W. Andersson ^b^, Maria Hahlin ^b,c^, Jonas Mindemark ^b^, Daniel Brandell ^b,*^, Jyh-Chiang Jiang ^a,*^

^a^ Department of Chemical Engineering, National Taiwan University of Science and Technology, Taipei 106, Taiwan.

^b^ Department of Chemistry - Ångström Laboratory, Uppsala University, Box 538, 75121, Uppsala, Sweden.

^c^ Department of Physics and Astronomy, Uppsala University, Box 516, 75120, Uppsala, Sweden.

Corresponding Author

*Jyh-Chiang Jiang ([jcjiang@mail.ntust.edu.tw](mailto:jcjiang@mail.ntust.edu.tw))

*Daniel Brandell ([daniel.brandell@kemi.uu.se](mailto:daniel.brandell@kemi.uu.se))


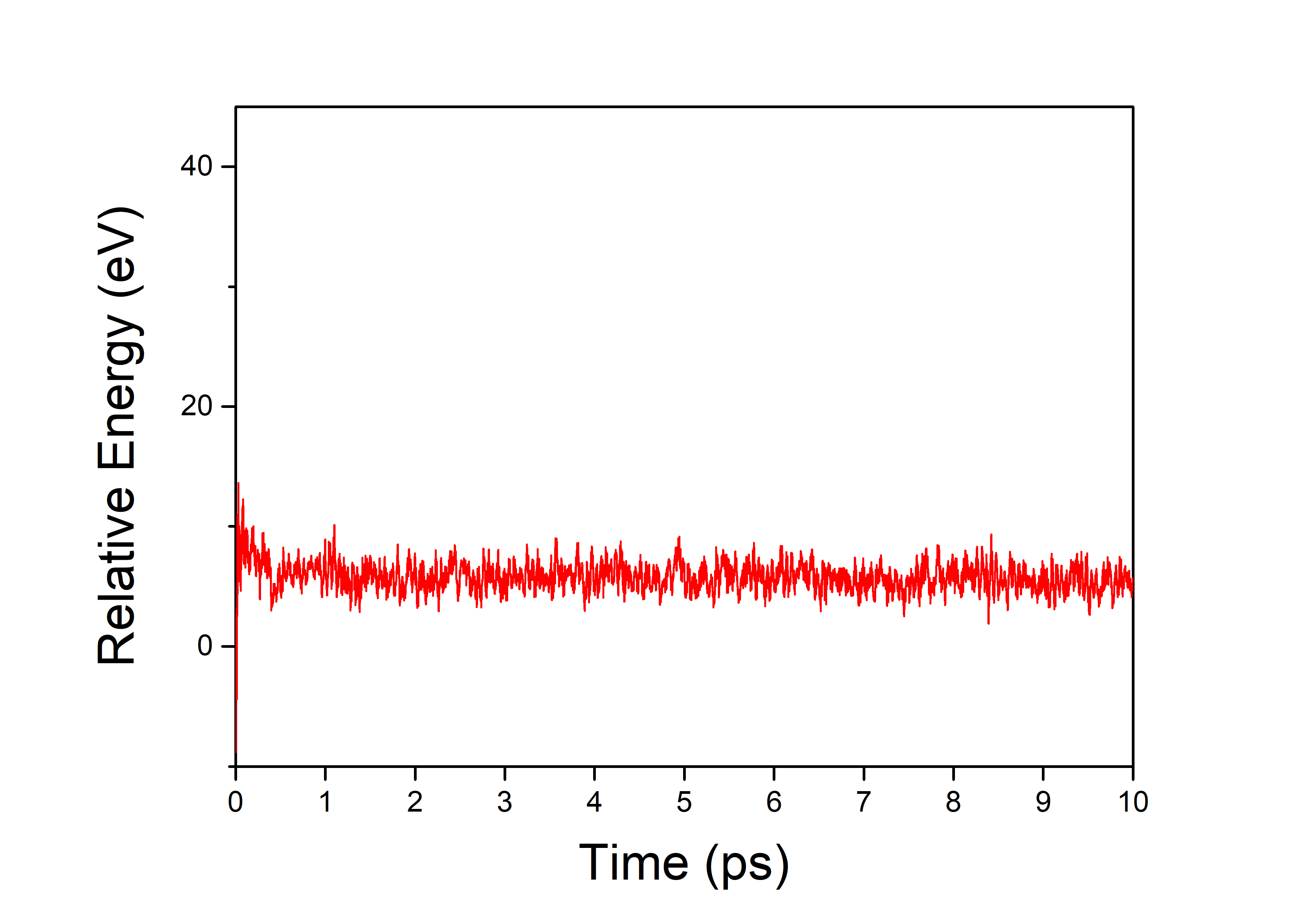


**Figure S1.** Energy plot over the simulation time for pure PEO.


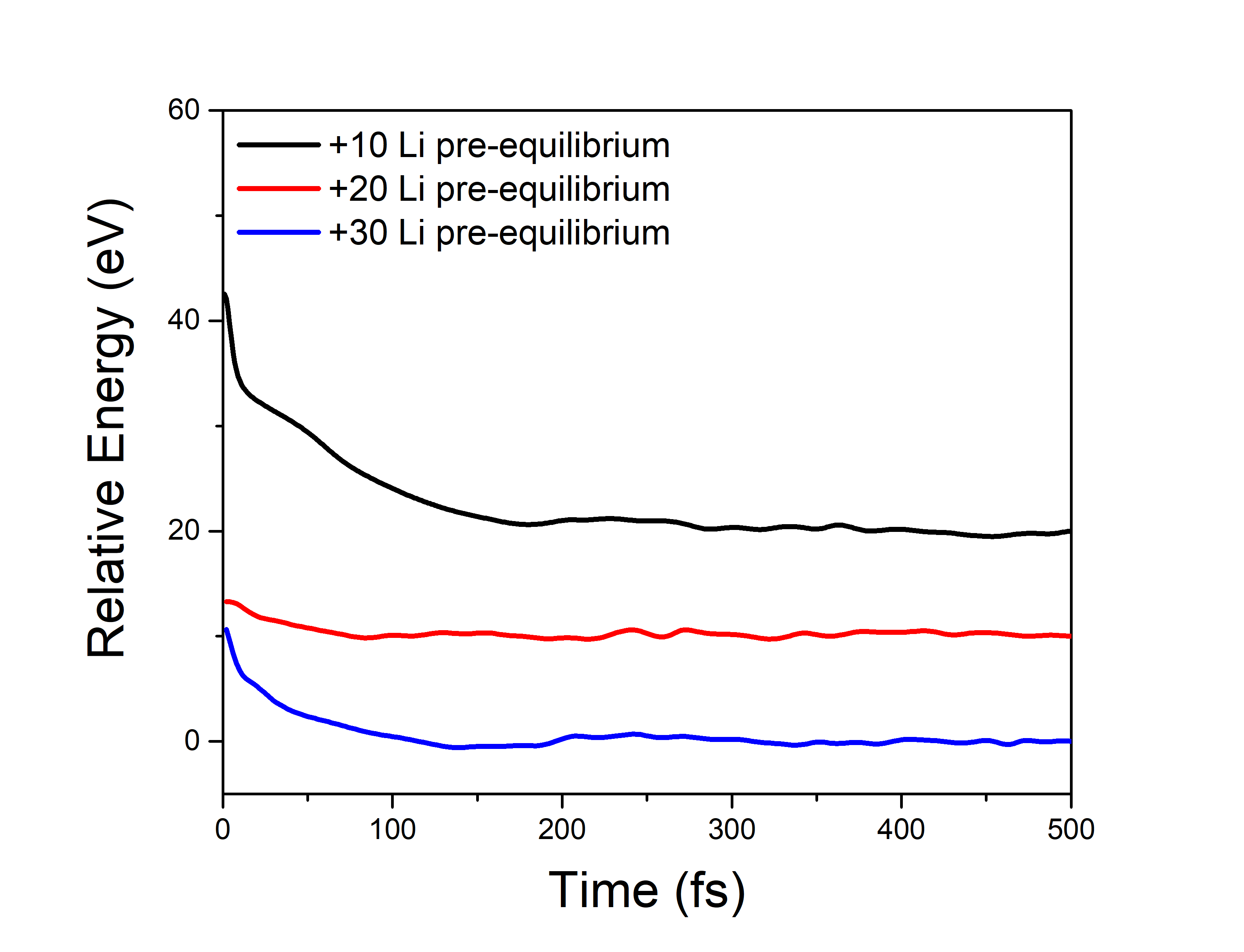


**Figure S2.** Energy plot over the simulation time for pre-equilibrium of PEO on Li (100) anode surface at different Li-nucleation stages.


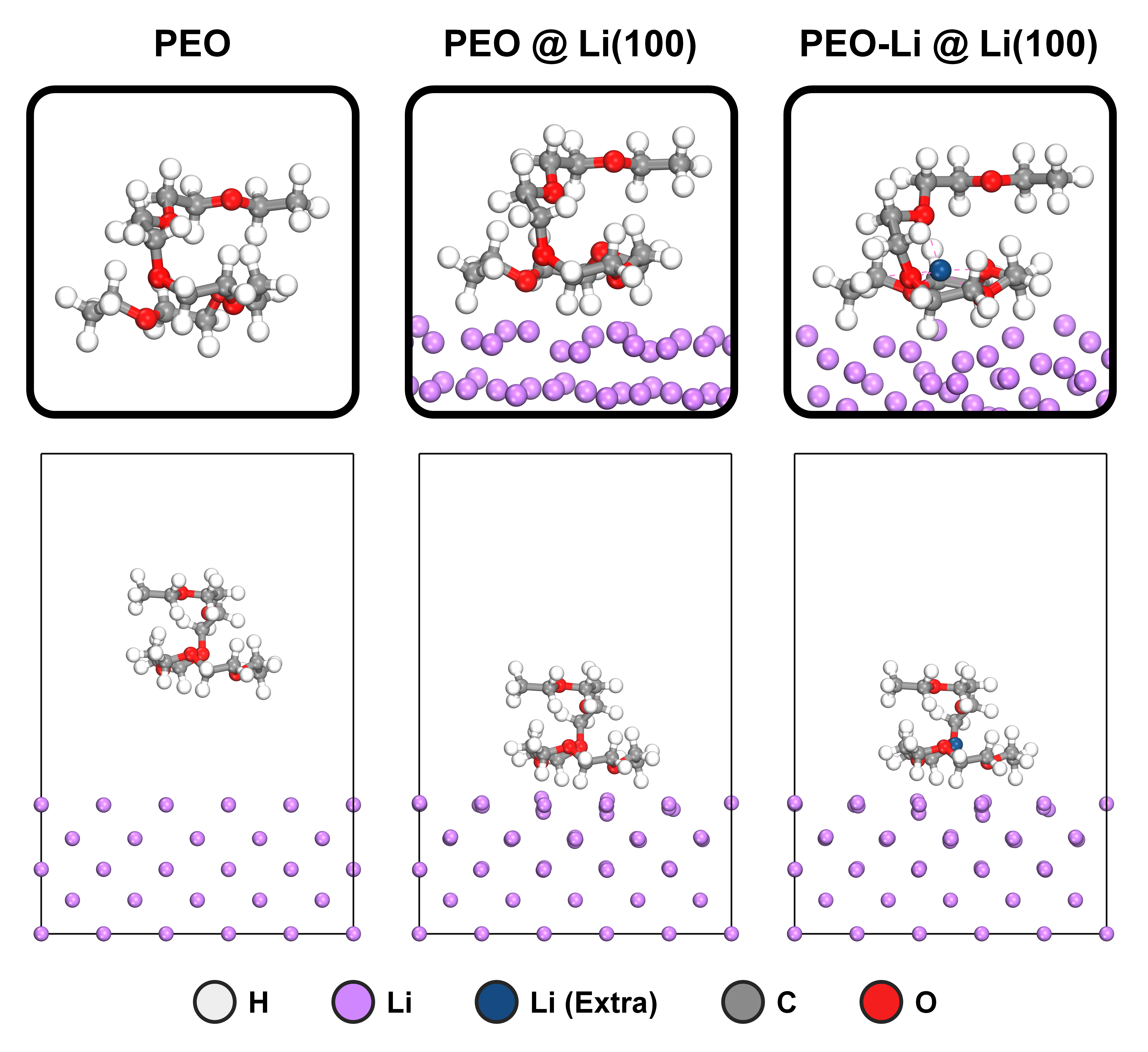
 **Figure S3.** Optimized structure of PEO in a vacuum layer, PEO adsorbed on the Li (100) surface, and PEO-Li complex adsorbed on the Li (100) surface.





**Figure S4.** Projected density of states (PDOS) of PEO oligomer in different conditions, including PEO in a vacuum layer, PEO adsorbed on the Li (100) surface, and the PEO-Li complex adsorbed on the Li (100) surface. Fermi levels of these systems are -1.484, -1.319, and -1.145 eV, respectively.


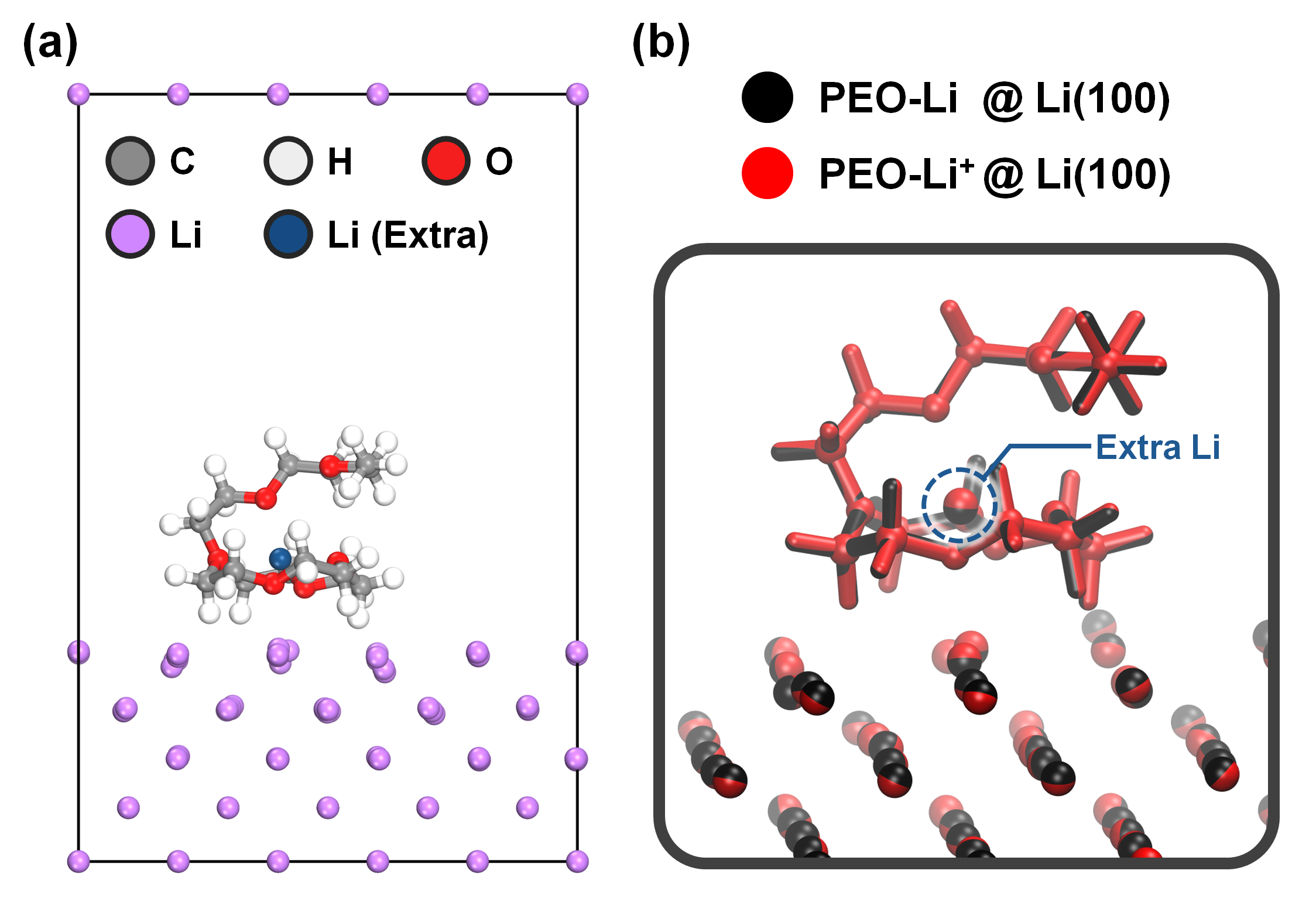


**Figure S5.** (a) The optimized structure of the PEO-Li^+^ complex adsorbed on the Li (100) surface and (b) the comparison of the geometry of systems in neutral and one positive charge conditions.


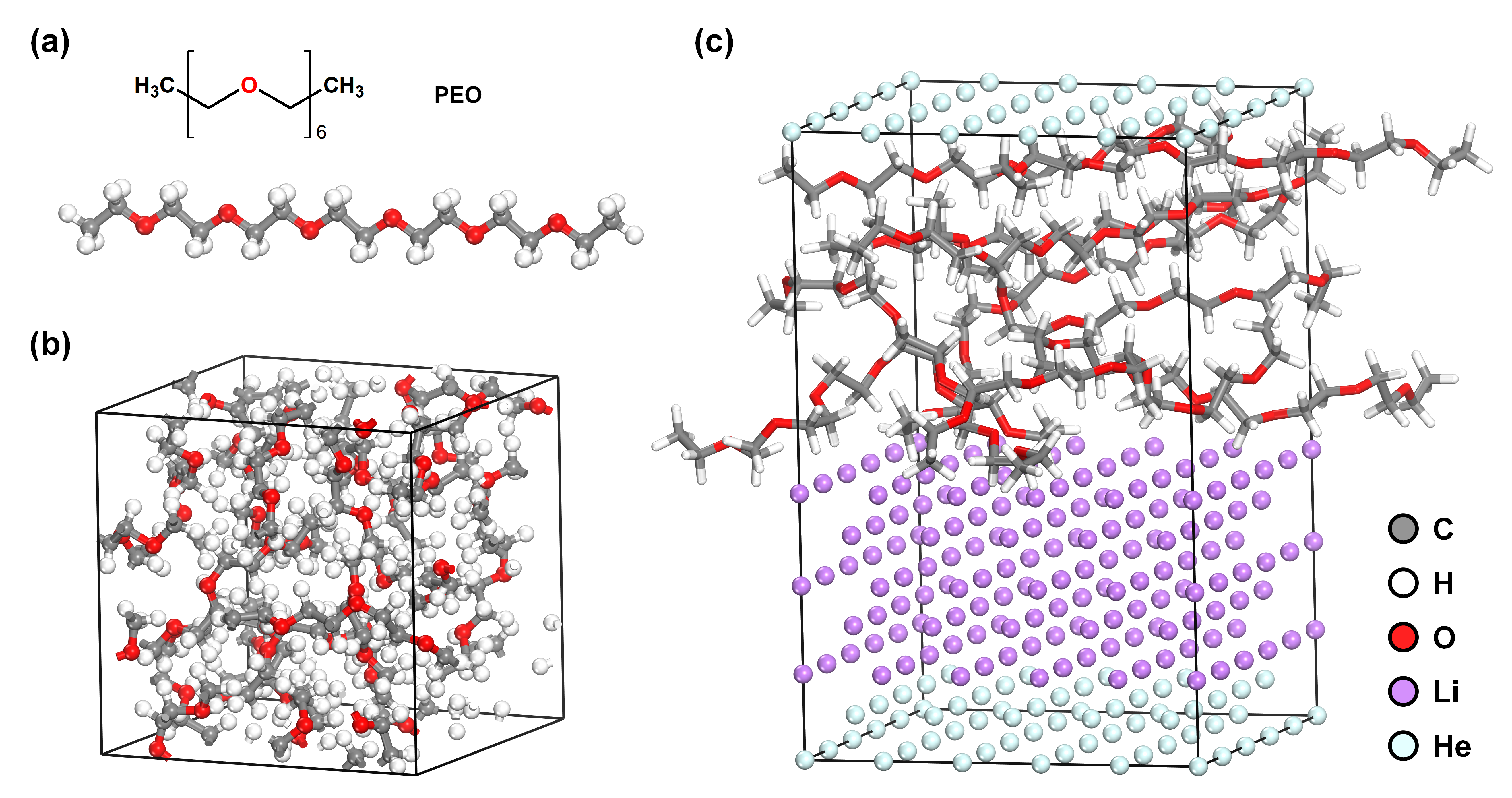


**Figure S6.** (a) The molecular structure of poly(ethylene oxide) (PEO) and initial configurations of the AIMD simulation box for (b) pure PEO (c) PEO on the Li (100) anode surface. The density of the pure PEO is 1.14 g cm^-3^.

**Table S1.** Identification of interfacial reactions of PEO on a Li-anode surface during Li-nucleation and the approximate time they occurred during AIMD simulations.

| **Systems** | **Time (fs)** | **Reactions** | **Products** |  |
| --- | --- | --- | --- | --- |
| 1^st^ PEO | 6890 | R-OC_2_H_4_O-R + 2Li → C_2_H_4_ + 2ROLi | ROLi, C_2_H_4_ | PEO decomposition |
|  | 7080 | C_2_H_4_ + 2Li → Li_2_C_2_H_4_ | Li_2_C_2_H_4_ | Li_2_C_2_H_4_ formation |
|  | 8050 | R-OC_2_H_4_O-R + 2Li → C_2_H_4_ + 2ROLi | ROLi, C_2_H_4_ | PEO decomposition |
|  | 11035 | C_2_H_4_ + 2Li → Li_2_C_2_H_4_ | Li_2_C_2_H_4_ | Li_2_C_2_H_4_ formation |
| 2^nd^ PEO | 8065 | R-OC_2_H_4_O-R + 4Li → Li_2_C_2_H_4_ + 2ROLi | ROLi, Li_2_C_2_H_4_ | PEO decomposition & Li_2_C_2_H_4_ formation |
| 3^rd^ PEO | 11200 | R-OC_2_H_4_O-R + 2Li → C_2_H_4_ + 2ROLi | ROLi, C_2_H_4_ | PEO decomposition |
|  | 11270 | C_2_H_4_ + 2Li → Li_2_C_2_H_4_ | Li_2_C_2_H_4_ | Li_2_C_2_H_4_ formation |
| 4^th^ PEO | 11485 | R-OC_2_H_4_O-R + 2Li → C_2_H_4_ + 2ROLi | ROLi, C_2_H_4_ | PEO decomposition |
